# Efficacy of Adherence-Enhancing Interventions for Immunosuppressive Therapy in Solid Organ Transplant Recipients: A Systematic Review and Meta-Analysis Based on Randomized Controlled Trials

**DOI:** 10.3389/fphar.2020.578887

**Published:** 2020-10-20

**Authors:** Yue-Xian Shi, Chun-Xia Liu, Fei Liu, Hai-Ming Zhang, Ming-Ming Yu, Yin-Hui Jin, Shao-Mei Shang, Ying-Xin Fu

**Affiliations:** ^1^ School of Nursing, Peking University, Beijing, China; ^2^ Department of Urinary Surgery, Peking University Third Hospital, Beijing, China; ^3^ School of Public Health, Peking University, Beijing, China; ^4^ Liver Transplantation Center, Clinical Center for Pediatric Liver Transplantation, National Clinical Research Center for Digestive Diseases, Beijing, China; ^5^ Center for Evidence-Based and Translational Medicine, Zhongnan Hospital of Wuhan University, Wuhan, China; ^6^ Center for Evidence-Based and Translational Medicine, Wuhan University, Wuhan, China; ^7^ Department of Kidney Transplantation, Tianjin First Center Hospital, Tianjin, China

**Keywords:** immunosuppression, organ transplantation, systematic review, meta-analysis, adherence

## Abstract

**Background:**

Immunosuppressant non-adherence is a widespread problem among solid organ recipients. With the newly published clinical trials, the randomized controlled trials (RCTs) based systematic review of adherence-enhancing interventions on immunosuppressant adherence in solid organ recipients has not been completed. In this systematic review and meta-analysis, we compared the efficacy of adherence-enhancing interventions versus routine intervention, as performed with RCTs, on immunosuppressant adherence in solid organ transplantation recipients.

**Methods:**

PubMed, Embase, Cochrane Library, CINAHL full text, and PsycINFO were searched from database inception to December 2019. This review was conducted following the PRISMA’s reporting guidelines and according to the principles recommended by Cochrane Handbook for Systematic Review.

**Results:**

The search yielded 10,479 articles. A total of 27 articles (26 studies) with 715 participants were included in our analysis. Results from the meta-analysis revealed that as compared with that of the routine intervention group, the rates of overall adherence, dosing adherence, and timing adherence were significantly increased within the adherence-enhancing intervention group, with the pooled risk ratio (RR) of overall adherence = 1.17, [95% confidence interval (CI): 1.07 to 1.28; p = 0.0006]; RR of dosing adherence = 1.21 (95% CI: 1.08 to 1.36, p = 0.001); RR of timing adherence = 1.16 (95% CI: 1.03 to 1.29, p = 0.01). There was a significantly increased adherence score in the adherence-enhancing intervention group; however, no statistical significance on the immunosuppressant blood concentration was found between the two study groups. Results obtained from a subgroup analysis shown interventions led by a multidisciplinary team, both the assessment time at 6 months and 12 months demonstrated a significantly increased adherence rate in the intervention group compared with the control group.

**Conclusions:**

The findings of this report indicate that clinicians (doctors and nurses) should maintain a long-term intervention protocol to ensure immunosuppressant adherence within solid organ transplant recipients. To accomplish this goal, we recommend a multidisciplinary team-led, comprehensive intervention approach combined with mobile health monitoring for the administration of an effective immunosuppressive therapy regimen.

## Introduction

Immunosuppressive therapy represents a life-long endeavor for solid organ transplant recipients. Unfortunately, these medication regimens often involve complex protocols, not only due to the number of pills required, but also to frequent dose adjustments based on blood level monitoring, side effects and rejection episodes.

As life-long immunosuppressive therapy is often critical for solid organ recipients and adherence to post-transplant immunosuppressants remains one of the most important factors for long-term allograft survival. Findings from several studies have indicated that immunosuppressant non-adherence is a pervasive problem among solid organ recipients. Non-adherence can be either deliberate or unintentional and include such events as not taking the medication as often as required, not in the exact dose and/or at the correct times (O’Carroll et al., 2006). The prevalence of immunosuppressant non-adherence can vary from 2 to 67% in solid organ transplant recipients (Martin and Gabardi, 2009; Zhang et al., 2019). The highest rate of immunosuppressant non-adherence was found in kidney transplant recipients with a prevalence of 36–55% (Gokoel et al., 2020), while that in adult heart transplant recipients is 34.1–41.1% (Leven et al., 2017) and 15–40% in liver transplants (Zhang et al., 2019).

Identification of effective intervention methods would be helpful to improve solid organ recipients’ immunosuppressant adherence. To date, considerable research has been devoted to systematic reviews and/or meta-analyses to determine whether interventions such as electronic monitoring feedback, pharmacist-led interventions, and cognitive education have a positive effect on immunosuppressant adherence. Results from two systematic reviews based on randomized controlled trials (RCTs) or prospective, retrospective, and cohort studies have found that adherence intervention could significantly improve immunosuppressive compliance within kidney transplant patients (Mathes et al., 2017; Zhu et al., 2017). Similarly, findings from a systematic review by Marcelino indicated that a psycho-educational intervention program exerted a positive impact on adherence in heart transplant patients (Marcelino et al., 2015). And a systematic review focusing on renal, heart, and liver transplant recipients revealed that a combination of interventions may be effective for long-term immunosuppressant adherence of solid organ recipients (De Bleser et al., 2009).

Based upon the results from these systematic reviews, the effectiveness of adherence-enhancing interventions on immunosuppressive therapy was expanded as achieved with adopting RCTs. In specific, three RCTs (Han et al., 2019; Levine et al., 2019; Geramita et al., 2020) adopted the mobile health or smartphone app as the main interventions to manage the immunosuppressive medication adherence in lung or kidney recipients, while Grady et al. conducted a pilot RCT study to determine whether a transition intervention could increase medication adherence in young heart transplant recipients (Grady et al., 2019).

Therefore, the new and RCTs based systematic review with meta-analysis would be necessary for further confirming the efficacy of adherence-enhancing interventions on immunosuppressant therapy in solid organ recipients. In this systematic review and meta-analysis, we compared the efficacy of adherence-enhancing interventions *versus* routine intervention, as performed with RCTs, on immunosuppressant adherence in solid organ transplantation recipients.

## Materials and Methods

This systematic review with meta-analysis was conducted following the PRISMA’s reporting guidelines (Maher, 2011) and according to the principles recommended by Cochrane Handbook for Systematic Review (Higgins and Green, 2008). We have registered this meta-analysis on PROSPERO and the information is available at: http://www.crd.york.ac.uk/PROSPERO/display_record.asp?ID=CRD42013006517. (registration number CRD42020172351).

### Search Methods

Computerized databases and manual literature searches were the two main data sources. We conducted a systematic search of full-text articles in the PubMed, Embase, Cochrane Library, CINAHL full text, and PsycINFO databases. MeSH or EMTREE terms together with text words were searched in the PubMed, Cochrane Library, and Embase databases, and the text words were adopted in other databases. All of the database searches were conducted from database inception to December 2019. The specific search strategy using PubMed as an example is in the **Supplementary Material** (Appendix 1, provided as online supplementary material). Manual searches of reference lists included additional relevant studies. We tried to identify unpublished studies by contacting experts who may have known about additional trials and retrieved the System for Information on Grey Literature database (http://opensigle.inist.fr/) to reduce publication bias.

### Inclusion and Exclusion Criteria

We selected all publications and screened studies if they met the following criteria:

#### Participants

Trials consisting of patients receiving immunosuppressive therapy after solid organ transplant, including liver, kidney, heart, lung, and pancreases, were included in this study. Immunosuppression medications were considered as any one, or combination, of the following: tacrolimus, cyclosporine, mycophenolate-mofetil, and sirolimus. These were selected according to the immunosuppression medications reported in the included studies.

#### Intervention

Interventions were aimed at enhancing immunosuppressant adherence and were classified as: (1) directors of intervention were nurse, pharmacist, transplant/coordinator physicians, and multidisciplinary, etc.; and (2) intervention programs included any one, or combination, of the following: using mobile health system/app, self-management intervention, computer based/internet based intervention, comprehensive intervention approach combined with mobile health monitoring, etc., which were selected according to the interventions reported in the included studies.

#### Comparators

Routine intervention (or interventions different from the adherence-enhancing intervention).

#### Outcome Measures

The outcome of this systematic review was the adherence to the immunosuppressive therapy. However, as a variety of outcome measures were used for assessing adherence, differences in the definition of adherence existed among the studies. When reviewing outcome measures from the included studies the following measures regarding immunosuppressive therapy adherence emerged:

1. Overall adherence rate: This mainly included the adherence rate if the original article directly reported this outcome. Otherwise, we used the taking adherence rate, dosing adherence rate, or self-reported adherence rate (assessed by questionnaires) sequentially. Immunosuppressant adherence was estimated using electronic monitoring, refill records, or patient self-reports. If more than one method was reported, we used electronic monitoring because of the available objective data and then the refill record and patient self-reports. The rate of taking, dosing, and timing adherence were pooled if the data were provided in the included studies. Taking adherence was the proportion of prescribed doses taken. Dosing adherence was the proportion of a patient’s actual immunosuppressant doses corresponding to the prescribed dosing regimen. Timing adherence was the proportion of prescribed doses taken within optimal inter-dose intervals.
2. Adherence score: When the score was used to express immunosuppressant adherence in the original studies, the score was pooled in our systematic review.
3. Immunosuppressant serum levels: We initially analyzed each study’s mean serum immunosuppressant concentrations and the immunosuppressant concentration rates within, above, and below the target level. The coefficient of variation (CV) and standard deviation (SD) of immunosuppressant concentrations were analyzed if the original study reported these outcomes.

#### Types of Studies

RCT design studies with full text and English literature were included.

### Study Selection

Two researchers independently screened all titles and abstracts and excluded studies that failed to satisfy the inclusion criteria. The full text of any published article that potentially satisfied the inclusion criteria was then reviewed to confirm its acceptance or rejection. Any disagreements about selection were resolved through discussion with a third reviewer. The list of potential studies was reviewed by two independent researchers. In cases of uncertainty regarding eligibility, a third reviewer was consulted.

### Quality Assessment

The methodological quality of the included studies was assessed by two independent reviewers. We followed the criteria of the Cochrane Collaboration risk-of-bias tool as guides. The quality items assessed were selection bias (random sequence generation and allocation concealment), performance bias (blinding of participants, personnel, and outcome assessors), attrition bias, measurement bias, reporting bias, and other bias.

### Data Extraction

The two researchers also developed a data extraction form, amended it as needed, and independently extracted the following data from each article: study characteristics (first author, publication year, and country), sample size, type of immunosuppression, outcomes and adherence assessment method, follow-up times, and interventions. We emailed study authors to obtain missing data or determine unclear information.

### Synthesis

The risk ratio (RR) was used as the count data effect size for the adherence rate, while the standard mean difference (SMD) was used to assess the effects of continuous outcomes. A RR >1 indicated a favorable intervention. When statistical heterogeneity (I^2^ ≥50%) was present among the studies, a random-effects model was used, otherwise, fixed-effects models were used. The source of statistical heterogeneity was evaluated using sensitivity analysis. Subgroup analyses by organ type, intervention director, follow-up time, adherence assessment method and intervention methods, and adherence assessment method were used to address clinical and methodological heterogeneity. Review Manager Version 5.3 (Copenhagen, Denmark) was used to synthesize and analyze the data.

## Results

### Study Selection

**Figure 1:**
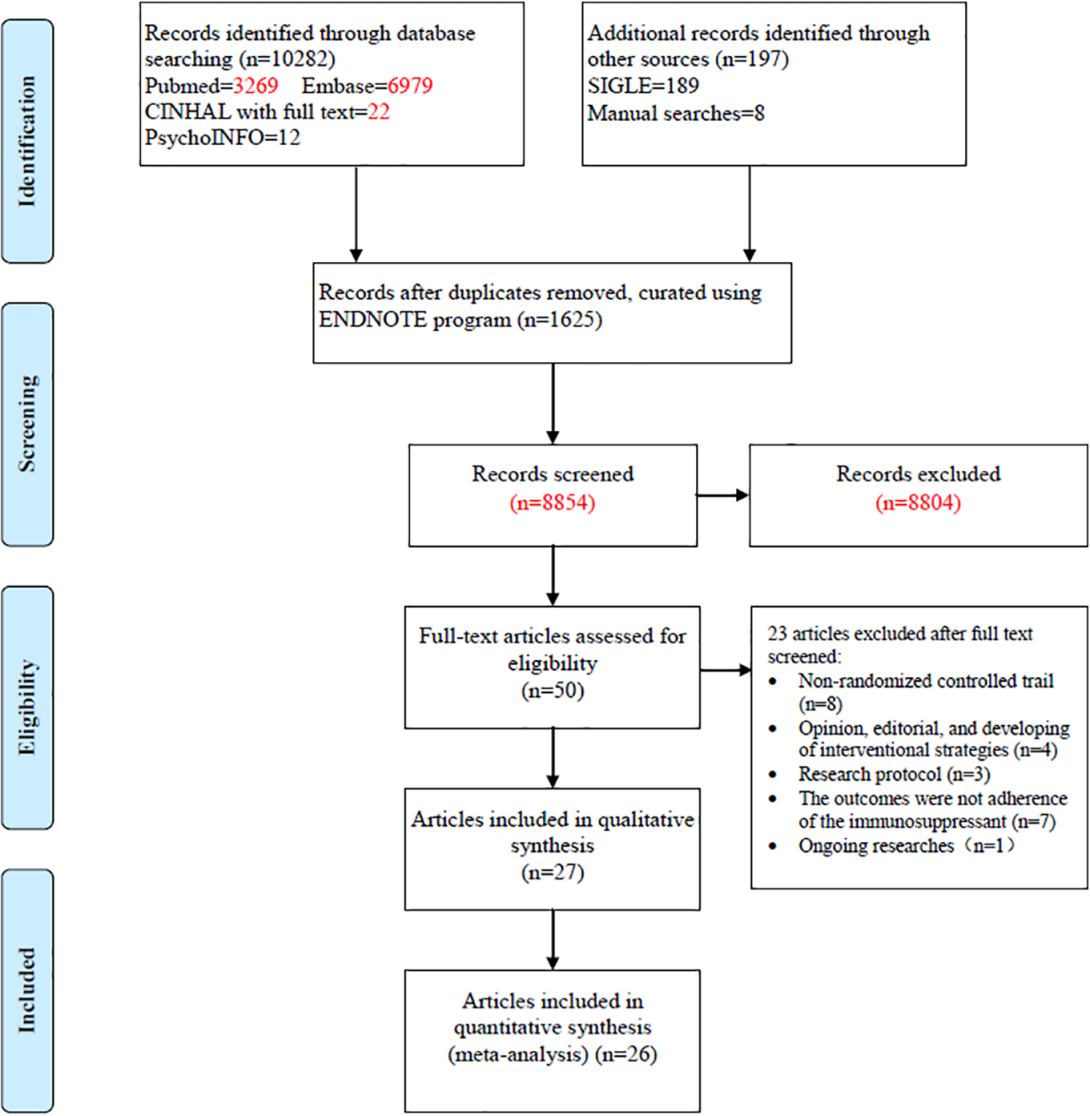
Selection of studies.

The search yielded 10,479 articles; 50 full-text articles underwent further assessment and 23 were excluded. The excluded articles included non-randomized controlled trials (n = 8), opinions, editorials, and interventional strategies (n = 4), research protocols (n = 3), outcomes failing to show immunosuppressant adherence (n = 7), and an ongoing study (n = 1). The remaining 27 published papers were then included in our systematic review (**Figure 1**) (Chisholm et al., 2001; Hardstaff et al., 2002; Hardstaff et al., 2003; De Geest et al., 2006; DeVito Dabbs et al., 2009; Klein et al., 2009; Russell et al., 2011; Chisholm-Burns et al., 2013; McGillicuddy et al., 2013; Suhling et al., 2014; Garcia et al., 2015; Bessa et al., 2016; Breu-Dejean et al., 2016; DeVito Dabbs et al., 2016; Henriksson et al., 2016; Cukor et al., 2017; Dobbels et al., 2017; Harrison et al., 2017; Reese et al., 2017; Rosenberger et al., 2017; Schmid et al., 2017; Foster et al., 2018; Grady et al., 2019; Han et al., 2019; Levine et al., 2019; Geramita et al., 2020; Russell et al., 2020). As participants in two studies (Hardstaff et al., 2002; Hardstaff et al., 2003) were from the same cohort, 26 studies were then finally included in the systematic review.

### Study Characteristics

Of the studies included studies, 14 were from the US (Chisholm et al., 2001; DeVito Dabbs et al., 2009; Russell et al., 2011; Chisholm-Burns et al., 2013; McGillicuddy et al., 2013; Suhling et al., 2014; DeVito Dabbs et al., 2016; Cukor et al., 2017; Reese et al., 2017; Rosenberger et al., 2017; Grady et al., 2019; Levine et al., 2019; Geramita et al., 2020; Russell et al., 2020) and Brazil (Bessa et al., 2016; Geramita et al., 2020), Germany (Klein et al., 2009; Schmid et al., 2017), Canada (Harrison et al., 2017; Foster et al., 2018), and the UK (Hardstaff et al., 2002; Hardstaff et al., 2003) had two studies, respectively. The others originated from France (Breu-Dejean et al., 2016), Switzerland (De Geest et al., 2006), Sweden (Harrison et al., 2017), Belgium (Dobbels et al., 2017), and Korea (Han et al., 2019) (**Table 1**). A total of 2,678 participants were enrolled in these studies. The transplants conducted in these 27 studies included 18 kidney (Chisholm et al., 2001; Hardstaff et al., 2002; Hardstaff et al., 2003; De Geest et al., 2006; Russell et al., 2011; Chisholm-Burns et al., 2013; McGillicuddy et al., 2013; Garcia et al., 2015; Bessa et al., 2016; Breu-Dejean et al., 2016; Henriksson et al., 2016; Cukor et al., 2017; Reese et al., 2017; Schmid et al., 2017; Foster et al., 2018; Han et al., 2019; Levine et al., 2019; Russell et al., 2020), 5 lung (DeVito Dabbs et al., 2009; Suhling et al., 2014; DeVito Dabbs et al., 2016; Rosenberger et al., 2017; Geramita et al., 2020), 2 solid organ (Dobbels et al., 2017; Harrison et al., 2017), a heart (Grady et al., 2019), and a liver recipient (Klein et al., 2009). The follow-up periods ranged from 6 weeks to 15 months. The intervention programs for the two groups are summarized in **Table S1** (provided as online **Supplementary Material**).

**Table 1:** The characteristics of the included studies.

Studies: Authors, Years, Country	Age (years) M ± SD median (IQR/range)	Sample size (I/C)	Type of transplantation	Immunosuppression	Adherence assessment
Bessa et al., 2016, Brazil	I: 45.7 ± 11.6 C: 43.1 ± 12.5	64/62	Kidney	Mycophenolate Azathioprine	Coefficient of variation %CV The percentage of patients who achieved tacrolimus target concentrations Dose-corrected whole blood tacrolimus trough concentrations Assessment of patient adherence using BAASIS
Breu-Dejean et al., 2016, France	I: 49.7 ± 11.6 C: 47.9 ± 12.8	55/55	Kidney	Cyclosporine Sirolimus Tacrolimus Mycophenolate mofetil Everolimus	Adapted questionnaire on adherence evaluation
Chisholm et al., 2001, USA	49.2 ± 10.2	12/12	Kidney	Cyclosporine Tacrolimus	Calculated Compliance rate (comparing patients’ monthly pharmacy refill records to the prescribed regimen documented in the patients’ medical records) Serum concentrations of cyclosporine and tacrolimus
Chisholm-Burns et al., 2013, USA	I: 52.78 ± 13.55 C: 51.32 ± 13.69	76/74	Kidney	Cyclosporine Tacrolimus	Calculated the immunosuppressant therapy adherence rate by pharmacy refill records
Cukor et al., 2017, USA	I: 49.1 (35–74) C: 55.6 (38–72)	15/18	Kidney	Tacrolimus	Medication adherence by phone pill count Tacrolimus trough levels
De Vito Dabbs et al., 2009, USA	I: 55 C: 57	15/15	Lung	Cyclosporine Tacrolimus	The Health Habits Assessment: determine post-transplant adherence in 10 areas: attending clinic appointments, completing blood work; monitoring home blood pressure and taking the primary immunosuppressant, etc. Reported of adherence by both patients and their primary family caregivers
De Vito Dabbs et al., 2016, USA	I: 62 (51–67) C: 62 (51–68)	99/102	Lung	Not involving	The Health Habits Survey was used to assess adherence to all elements of the medical regimen (e.g., taking medications, attending clinic appointments, completing lab work) Reported of adherence by both patients and their primary family caregivers
De Geest et al., 2006, Switzerland	45.6 ± 1.2	6/12	Kidney	Cyclosporine Mycophenolae-Mofetil Tacrolimus Sirolimus	Adherence to immunosuppressive regimen was measured by electronic monitor
Dobbels et al., 2017, Belgium	I: 56.1 ± 11.7 C: 56.2 ± 11.8	103/102	Heart, Liver and Lung	Tacrolimus	The ABC taxonomy for medication adherence by electronic monitor Poor implementation in relation to medication taking BAASIS for adherence of the immunosuppressant
Foster et al., 2018, Canada	I: 15.8 (13.3–17.5) C: 15.5 (13.2–17.4)	72/88	Kidney	Tacrolimus	Taking adherence and timing adherence as measured using electronic monitoring. Standard deviation of tacrolimus trough levels Self-reported adherence: Medical Adherence Measure Medication Module (MAM-MM)
Garcia et al., 2015, Brazil	I: 46.00 ± 14.1 C: 49.29 ± 12.1	55/56	Kidney	Cyclosporine Sirolimus Tacrolimus Mycophenolate	Adherence of immunosuppressive therapy: using Immunosuppressant Therapy Adherence Scale Adherence rate Serum levels of immunosuppressant drugs
Geramita et al., 2020, USA	I: 56.2 ± 12.3 C: 56.0 ± 14.2	47/58	Lung	Unclear	Health Habits Survey: taking the primary immunosuppressant, taking other medications; Non-adherence was assessed by combination of patient and family caregiver report
Grady et al., 2019, USA	I: 21.3 ± 3.2 C: 21.5 ± 3.3	43/45	Heart	Tacrolimus Mycophenolic acid	Tacrolimus levels Self-report of adherence
Han et al., 2019, Korea	I: 45 (35–54) C: 43 (30–52)	70/66	Kidney	Tacrolimus	1) Medication taking adherence, dosing adherence, timing adherence, and drug holidays by electronic monitoring 2) Self-reported rate of non-adherence
Hardstaff et al., 2002, United Kingdom	Not mentioned	75/25	Kidney	Unclear	Multidimensional Adherence by electric monitor
Hardstaff et al., 2002, United Kingdom	Not mentioned	23/25	Kidney	Unclear	The compliance in the time period
Harrison et al., 2017, Canada	I: 48.1 ± 13.7 C: 49.6 ± 12.4	126/120	Heart, kidney, kidney-pancreas, liver, lung, liver-kidney	Tacrolimus Cyclosporine	For each immunosuppressant, the number of doses missed or taken late in the last week was collected *via* patient self-report. Classification System
Henriksson et al., 2016, Sweden	I: 48.1 ± 13.7 C: 49.6 ± 12.4	40/40	Kidney	Tacrolimus Cyclosporine Sirolimus	Immunosuppressive adherence: Patients skipped their medicine dose, the number of missed doses; outpatient follow-up visits recorded by electronic medication dispenser
Klein et al., 2009, Germany	I: 52.8 (28–65) C: 50.1 (30–63)	24/24	Liver	Not mentioned	Patients’ compliance with the immunosuppressive therapy was assessed by medication event monitoring systems Calculated the compliance rates Immunosuppressant serum concentrations Patients were asked in writing how often they forgot to take a dose of their immunosuppressant during the last 4 weeks.
Levine et al., 2019, USA	I (Mobile app): 52 I (Watch/Mobile App User): 50 C (No App User): 53	38/20/50	Kidney	Tacrolimus Mycophenolic	Immunosuppressive medication adherence: coefficient of variability ¼ (SD/mean tacrolimus)*100
McGillicuddy et al., 2013, USA	I: 42.44 C: 57.6	9/10	Kidney	Not mentioned	Calculation of medication adherence score by the data form Prototype mHealth System
Reese et al., 2017, USA	I1: 50 ± 12 I2: 50 ± 11 C: 49 ± 11	40/39/38	Kidney	Tacrolimus	Adherence according to wireless Electronic pill bottle Blood Trough Concentrations Self-rated Adherence using the BAASIS adherence questionnaire Pharmacist Assessment
Rosenberger et al., 2017, USA	I:57 ± 13 C: 58 ± 14	96/102	Lung	Not mentioned	Self-report adherence Collateral (family caregiver) report using the Health Habits Assessment instrument
Russell et al., 2011, USA	I: 55 C: 44	8/7	Kidney	Not mentioned	Medication non-adherence measured by Medication Event Monitoring System (adherence score)
Russell et al., 2020, USA	I: 53.0 ± 11.2 C: 50.7 ± 9.7	45/44	Kidney	Not mentioned	Average 6‐month immunosuppressive medication adherence rate by the Medication Event Monitoring System SmartCap Adherence at 12 months;
Schmid et al., 2017, Germany	I: 46 (18–59) C: 51 (19–66)	23/23	Kidney Living donor	1) Tacrolimus 2) Mycophenolic acid	1) Composite adherence score: by using BAASIS 2) Composite adherence percentage: Collateral reports (physicians, nurses) and the target tacrolimus trough levels
Suhling et al., 2014, USA	I: 52 (35.9, 57.6) C: 45 (33.3, 53.9)	32/32	Lung	Cyclosporine Tacrolimus	BAASIS for medication intake adherence Percentage of calcineurin inhibitor trough levels Physicians’ valuation of adherence

BAASIS, Basel assessment of adherence with immunosuppressive medication scales; M, mean; SD, standard deviation; IQR, interquartile range; I, intervention group; C, control group.

### Methodological Quality

**Figure 2:**
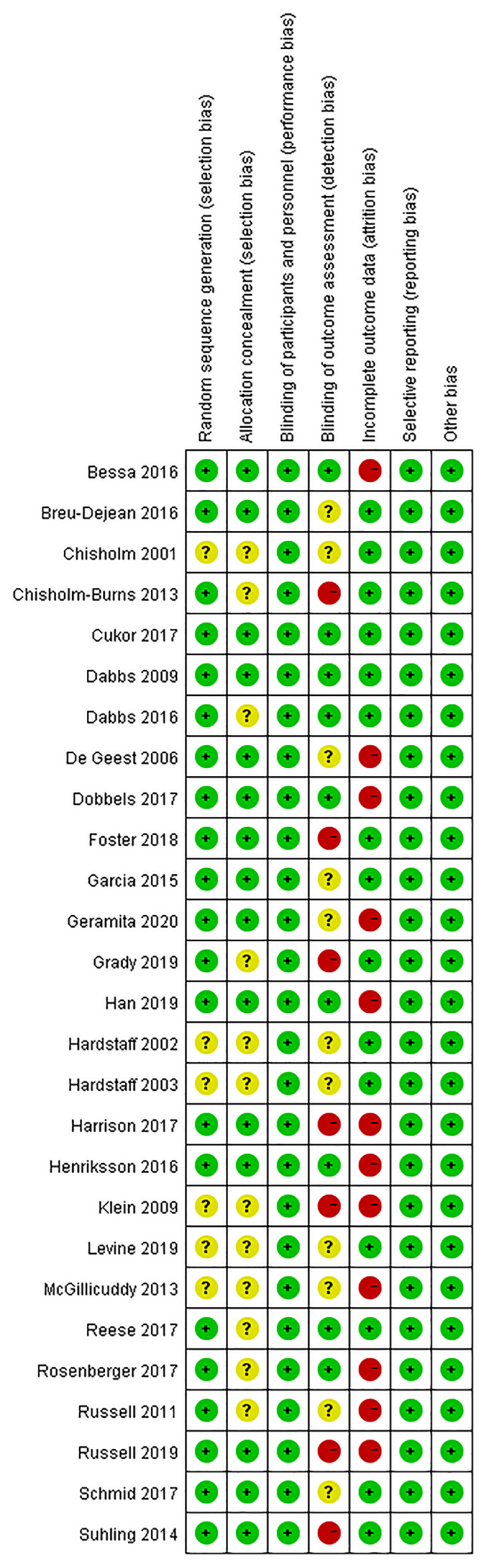
Risk of bias graph.

**Figure 3:**
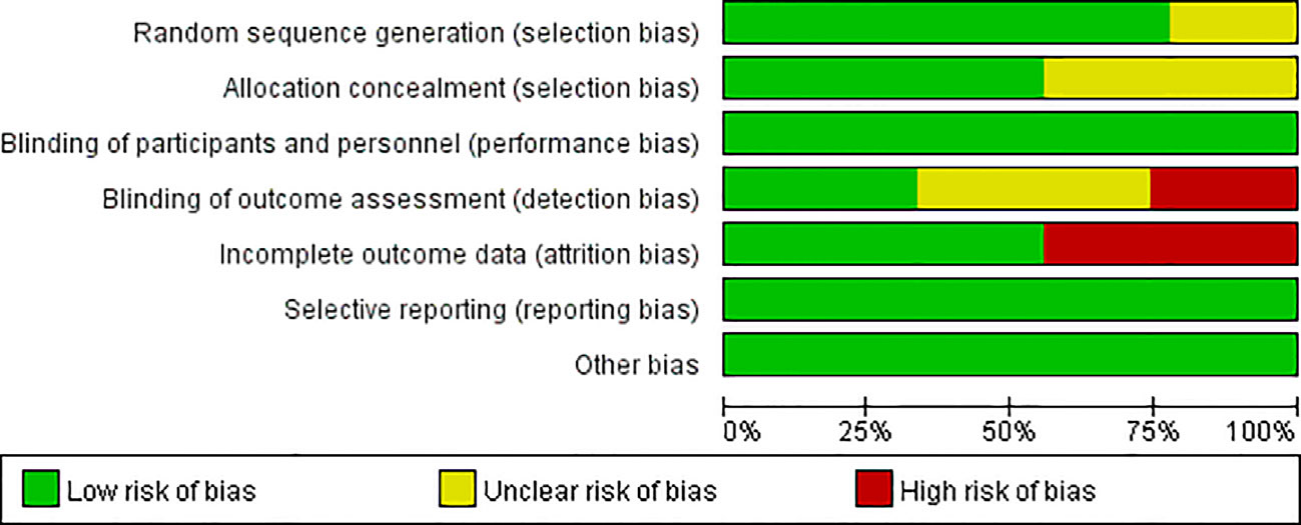
Risk of bias summary.

**Figures 2**, **3** present the results of the risk of bias assessment. As it was difficult to blind personnel/participants in interventions to improve adherence, blind personnel/participants in all of the included studies were assessed as a low risk of bias.

### Effect of Intervention

Immunosuppressive Therapy Adherence as Assessed by Adherence Rate

**Figure 4:**
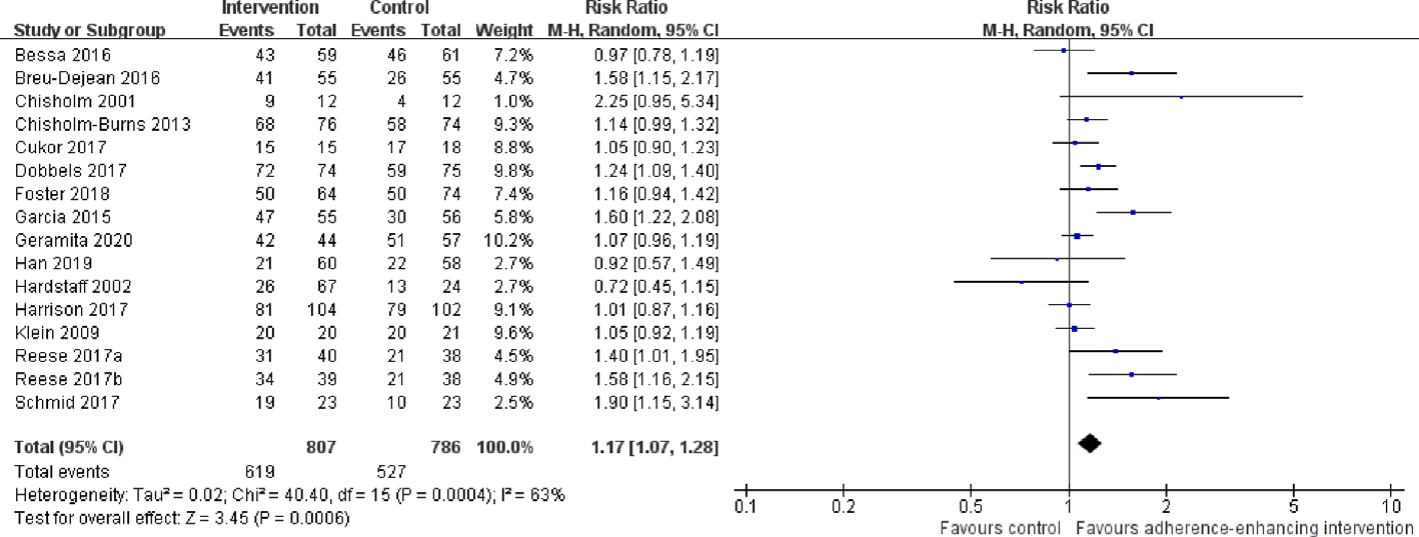
Forest plot of overall adherence rate. Risk Ratio (RR) with 95% confidence interval (CI) between the adherence enhancing intervention group and routine intervention groups.

**Figure 5:**
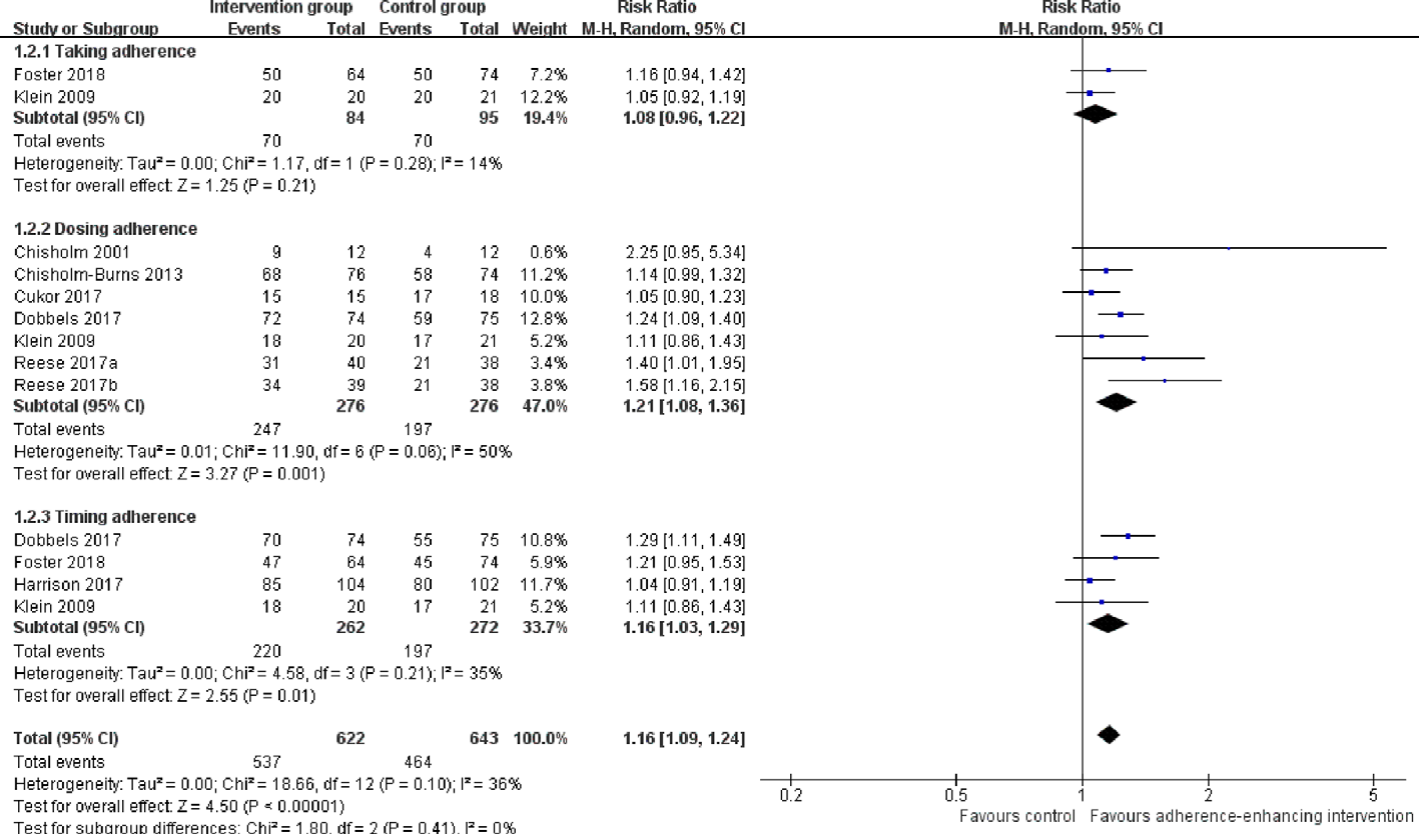
Forest plot of different adherence rate. Risk Ratio (RR) with 95% confidence interval (CI) between the adherence enhancing intervention group and routine intervention groups.

1. Overall adherence rate: A total of 19 RCTs (Chisholm et al., 2001; Hardstaff et al., 2002; Hardstaff et al., 2003; De Geest et al., 2006; Klein et al., 2009; Chisholm-Burns et al., 2013; Suhling et al., 2014; Garcia et al., 2015; Bessa et al., 2016; Breu-Dejean et al., 2016; Cukor et al., 2017; Dobbels et al., 2017; Harrison et al., 2017; Reese et al., 2017; Rosenberger et al., 2017; Schmid et al., 2017; Foster et al., 2018; Han et al., 2019; Geramita et al., 2020) reported immunosuppressant adherence rate, and we were able to extract 16 sets of analyzable data from 15 RCTs (Chisholm et al., 2001; Hardstaff et al., 2002; Klein et al., 2009; Chisholm-Burns et al., 2013; Garcia et al., 2015; Bessa et al., 2016; Breu-Dejean et al., 2016; Cukor et al., 2017; Dobbels et al., 2017; Harrison et al., 2017; Reese et al., 2017; Schmid et al., 2017; Foster et al., 2018; Han et al., 2019; Geramita et al., 2020) with 1,593 participants in the meta-analysis. A statistically significant heterogeneity was observed among these studies (I^2^ = 63%, p = 0.0004). Results from the meta-analysis showed that participants in the adherence-enhancing intervention groups showed significantly increased adherence rate as compared with those in the routine intervention groups with a pooled RR = 1.17 [95% confidence interval (CI): 1.07 to 1.28; p = 0.0006] (**Figure 4**). We did not include four RCTs in the pooled analysis as no analyzable data (Hardstaff et al., 2003; De Geest et al., 2006; Suhling et al., 2014) were available or they included comprehensive adherence rate in their analyses (including immunosuppressants, attending clinic appointments, monitoring vital signs) (Rosenberger et al., 2017), which were then not appropriate for meta-analysis. Details regarding these studies are summarized in **Table S2** (provided as online **Supplementary Material**).
2. The rate of taking, dosing, and timing adherence: There were two RCTs reporting taking (Klein et al., 2009; Foster et al., 2018), six reporting dose (Chisholm et al., 2001; Klein et al., 2009; Chisholm-Burns et al., 2013; Cukor et al., 2017; Dobbels et al., 2017; Reese et al., 2017), and four reporting timing adherence (Klein et al., 2009; Dobbels et al., 2017; Harrison et al., 2017; Foster et al., 2018). The meta-analysis showed that the dose and timing immunosuppressant adherence rates were significantly improved in participants receiving adherence-enhancing interventions, with pooled RR of dosing adherence = 1.21 (95% CI: 1.08 to1.36, p = 0.001) and RR of timing adherence = 1.16 (95% CI: 1.03 to 1.29, p = 0.01) (**Table 2** and **Figure 5**). Although there were three RCTs adopting taking (Hardstaff et al., 2003; De Geest et al., 2006; Han et al., 2019), two dose (Hardstaff et al., 2003; Han et al., 2019), and two timing adherence (De Geest et al., 2006; Han et al., 2019), no available data or unsuitable data were employed for the meta-analysis. Details on these outcomes are summarized in **Table S2**.
3. Adherence rate assessed by questionnaires: Thirteen RCTs (DeVito Dabbs et al., 2009; Suhling et al., 2014; Garcia et al., 2015; Bessa et al., 2016; Breu-Dejean et al., 2016; DeVito Dabbs et al., 2016; Dobbels et al., 2017; Reese et al., 2017; Schmid et al., 2017; Foster et al., 2018; Grady et al., 2019; Han et al., 2019; Geramita et al., 2020) assessed adherence rates using questionnaires. We were able to extract six sets of analyzable data from 5 RCTs to combine within our analyses (Bessa et al., 2016; Breu-Dejean et al., 2016; Reese et al., 2017; Grady et al., 2019; Han et al., 2019). Results from this meta-analysis failed to achieve statistical significance between the two groups, with RR = 1.16 (95% CI: 0.94 to 1.44, p = 0.17) (**Table 2** and **Figure S1**). Five RCTs (DeVito Dabbs et al., 2009; DeVito Dabbs et al., 2016; Schmid et al., 2017; Grady et al., 2019; Geramita et al., 2020) reported comprehensive adherence rates, which included not only immunosuppressant medications, but also clinic attendance and health monitoring, while three (Suhling et al., 2014; Dobbels et al., 2017; Foster et al., 2018) others did not provide analyzable data or median and mean, thus precluding the possibility for combining these statistics with other data. A summary of these studies is presented in **Table S3** (provided as online **Supplementary Material**).

Blood Immunosuppressant Concentration

1. Tacrolimus level: Six RCTs (Garcia et al., 2015; Bessa et al., 2016; Cukor et al., 2017; Reese et al., 2017; Schmid et al., 2017; Grady et al., 2019) reported tacrolimus blood levels and the mean and standard differences were extracted from five (Garcia et al., 2015; Bessa et al., 2016; Cukor et al., 2017; Reese et al., 2017; Grady et al., 2019). A total of 469 patients participated in these five studies and the fixed-effects model was adopted because of an accepted heterogeneity among these studies (I^2^ = 0%, p = 0.90). The pooled analysis showed that there was no significant difference between the two groups, with pooled SMD = −0.04 (95% CI: −0.23 to 0.14, p = 0.63) (**Table 2** and **Figure S2**). The study of Schmid et al. (2017) was not included in this analysis as a comprehensive adherence rate (combination of tacrolimus trough levels, collateral reports, and self-reported adherence rates) was used for analysis in that study (**Table S3**).
2. Coefficient of variation and standard deviation for blood tacrolimus or cyclosporine levels: The coefficient of variation and standard deviation were also used to estimate immunosuppressive therapy adherence in three (Bessa et al., 2016; Reese et al., 2017; Levine et al., 2019) and two RCTs (Harrison et al., 2017; Foster et al., 2018) respectively. Meta-analyses were not conducted because of the small number of studies or insufficient data in individual studies (**Table S4**, provided as online **Supplementary Material**).
3. Rate of immunosuppressant blood levels within the target range: This variable was reported in seven RCTs (Chisholm et al., 2001; Klein et al., 2009; Suhling et al., 2014; Bessa et al., 2016; Harrison et al., 2017; Reese et al., 2017; Grady et al., 2019), and the data from three studies (Bessa et al., 2016; Harrison et al., 2017; Grady et al., 2019) were combined. We used the random-effects model because of significant heterogeneity among these studies (I^2^ = 63%, p = 0.07). Results of this meta-analysis found no significant group differences on the rate of immunosuppressant blood levels within the target range, with the pooled RR = 1.00 (95% CI: 0.69 to 1.46, p = 1.00) (**Table 2** and **Figure S3**). The four other studies were not included in the pooled analysis because they reported both the tacrolimus and cyclosporine target range (Chisholm et al., 2001), adopting the mean, standard difference, or median and interquartile as statistics (Suhling et al., 2014; Reese et al., 2017) and using the percentage of blood test times meeting the target value (Klein et al., 2009). Bessa (Bessa et al., 2016) and Klein (Klein et al., 2009) analyzed the rate of immunosuppressant blood levels above or below the target range, and the results of meta-analysis did not find statistical significance between the groups (**Table S4** and **Figure S3**).
4. Adherence Score: Four RCTs (Russell et al., 2011; McGillicuddy et al., 2013; Garcia et al., 2015; Russell et al., 2020) assessed immunosuppressive therapy using adherence score. These studies included 232 participants and the random-effects model was adopted because of significant heterogeneity among these studies (I^2^ = 69%, p=0.02). Significant differences in the pooled analysis were obtained between the two groups, with the pooled SMD = 1.14 (95% CI: 0.52 to 1.75, p = 0.0003) (**Table 2** and **Figure S4**).

**Table 2:** The results of meta-analysis for the effectiveness of adherence enhancing interventions on adherence of immunosuppressive therapy.

	No. of included studies	Sample size in meta-analysis	Heterogeneity		Effect sizes			
			*I^2^*	*p*	*RR/SMD*	*95%CI*	*Z*	*p*
**Adherence rate**								
Overall adherence rate	15	1,593	63%	0.0004	1.17	1.07, 1.28	3.45	0.0006
Taking adherence rate	2	179	14%	0.28	1.08	0.96, 1.22	1.25	0.21
Doing adherence rate	6	552	50%	0.06	1.21	1.08, 1.36	3.27	0.001
Timing adherence rate	4	534	35%	0.21	1.16	1.03, 1.29	2.55	0.01
**Adherence rate by organ type**								
Kidney	11	1,096	64%	0.001	1.23	1.08, 1.41	3.06	0.002
Others	4	497	43%	0.16	1.10	1.00, 1.22	2.02	0.04
**Adherence rate by interventional director**								
Multidisciplinary	5	560	28%	0.23	1.45	1.25, 1.67	5.02	<0.00001
Pharmacist	5	541	25%	0.26	1.07	0.96, 1.19	1.23	0.22
Other	5	492	54%	0.07	1.08	0.95, 1.22	1.18	0.24
**Adherence rate by different assessment method**								
Electronic monitor	7	793	47%	0.07	1.16	1.04, 1.30	2.62	0.009
Self-reported or collateral report	7	822	71%	0.002	1.18	1.02, 1.36	2.26	0.02
Others	2	79	92%	0.0004	1.39	0.54, 3.55	0.68	0.49
**Adherence rate by intervention way**								
Including mobile health	3	265	66%	0.05	1.19	0.83, 1.70	0.94	0.35
Electronic medication only	2	246	75%	0.02	1.21	0.79, 1.84	0.88	0.38
Others	9	1,039	62%	0.007	1.17	1.05, 1.31	2.93	0.003
**Adherence rate by follow-up time**								
≤3-month	7	866	69%	0.002	1.13	0.98, 1.30	1.73	0.08
>3 to ≤6-month	4	527	46%	0.14	1.22	1.05, 1.42	2.60	0.009
≥12-month	5	562	46%	0.12	1.13	1.02, 1.25	2.37	0.02
**Adherence rate assessed by questionnaire**	5	602	75%	0.001	1.16	0.94, 1.44	1.37	0.17
**Immunosuppressant levels**								
Tacrolimus concentration	5	469	0	0.90	-0.04	−0.23, 0.14	0.49	0.63
Within target	3	303	63%	0.07	1.00	0.69, 1.46	0.00	1.00
Above target	2	321	84%	0.01	0.68	0.27, 1.72	0.81	0.42
Below target	2	321	93%	0.0001	0.64	0.06, 6.52	0.38	0.71
**Adherence score**	4	232	69%	0.02	1.14	0.52, 1.75	3.61	0.0003

RR, risk ratio; SMD, standard mean difference; CI, confidence interval.

### Subgroup Analysis and Sensitivity Analysis

Results from the sensitivity analysis did not reveal any changes in the results when excluding studies with a high risk of bias or a large effect size to test the robustness of the results. Subgroup analyses were separated by organ transplant type (kidney, lung, etc.), intervention director (pharmacist, nurse, or coordinator), follow-up time, adherence assessment method (self-reported or electronic monitoring), and intervention methods. With respect to the organ type, the effect of adherence-enhancing intervention on adherence rate included 12 studies with kidney recipients, one with liver, one with lung, and two studies included several organ types (e.g. heart/lung/kidney/liver-kidney/kidney-pancreases). Due to the small number of studies on liver, lung, heart or combined organ recipients, these data were pooled to generate two subgroups for comparison (kidney and others). Results of the meta-analysis for these two subgroups indicated that the adherence rate was significantly increased in both groups in response to the adherence-enhancing intervention. Multidisciplinary-led interventions displayed a statistically increased adherence rate after receiving the adherence-enhancing intervention (**Table 2** and **Figures S5**, **S6**). When assessed at a ≥6 month follow-up period, a significantly increased immunosuppressive therapy adherence rate was present in the intervention group (**Table 2** and **Figure S7**). The results also found that the outcomes whether assessed using electric monitoring or self-reporting displayed significant differences between the groups (**Table 2** and **Figure S8**). We did not find that intervention together with mobile health or adopting electronic monitoring only could be more effective than control groups (**Table 2** and **Figure S9**).

## Discussion

### Summary of Main Findings

Immunosuppressive therapy is critical for solid organ transplant patients and poor adherence to immunosuppressive therapy can negatively impact the long-term outcomes of these patients. Accordingly, improving drug compliance represents an important component for the long-term care of these patients after surgery. In this report, we provide the first systematic review and meta-analysis on the effects of adherence interventions as based on RCT studies. Our meta-analysis revealed that adherence-enhancing interventions can result in significant increases in total adherence, medication dosing, and timing adherence rates, as well as improvements in immunosuppressive therapy adherence scores. Patients receiving kidney transplants have been shown to benefit from interventions and a multidisciplinary-led intervention provided an effective approach to educate and monitor patients. These benefits resulting from interventions have been demonstrated under conditions where outcomes were assessed using either electric monitoring or self-reporting. Moreover, effects of adherence-enhancing intervention persist, as determined after 6 months post-intervention and were sustained for a year. At present, there is insufficient evidence to assess which type of intervention (mobile health, cognitive, or behavioral) may be maximally effective.

Several systematic reviews have focused on the effectiveness of interventions such as behavioral and cognitive interventions or medication knowledge improvement on adherence of immunosuppressive therapy patients. Of these, only one study pooled the outcomes of their findings. The results of this study demonstrated that adherence rate was significantly increased in kidney transplant patients receiving intervention programs designed to increase their immunosuppressive adherence as compared with that of a control group (Zhu et al., 2017). A systematic review from the Joanna Briggs Institute (JBI) also provided weak evidence supporting the effectiveness of interventions to manage adherence in heart or heart-lung transplantation patients (Guimarães Marcelino and da Cruz, 2013). De Bleser et al. examined the effectiveness of adherence-enhancing interventions for solid organ (renal, heart, and liver) transplant recipients, and the results of their systematic review indicated that a combination of interventions in a team approach might be effective over the long term (De Bleser et al., 2009).

Adherence to immunosuppressive interventions implies not only intake of medicines, but also taking the correct dose of immunosuppressants at the appropriate time. A number of approaches are currently used to estimate adherence to immunosuppressive interventions. For example, calculating adherence rates have used the data from electronic monitoring, pill accounts, self-reports or collateral reports, measurement of blood immunosuppressant concentrations, and self-reported questionnaires. While electronic monitoring is considered as the gold standard for assessing non-adherence, it may not be feasible in daily clinical practice. Self-reporting alone might cause over- or under-reporting (Foster et al., 2018), so the information derived from various sources (self-reports and collateral reports) is more recommended than single-measurement methods (De Geest et al., 2006; Foster et al., 2018). Blood immunosuppressant concentrations, especially tacrolimus levels, were also used to reflect adherence in many studies, but may be influenced by variations in metabolism and other confounding factors (Burra et al., 2011). Results of our meta-analysis demonstrated that adherence-enhancing intervention could be effective for producing adherence to immunosuppressive therapy, as indicated from results obtained using electronic monitoring and self-reports and/or collateral adherence reports. We did not find a significant difference in outcomes related to blood immunosuppressant levels between the groups, possibly due to the small sample size of studies in the pooled analysis. With respect to self-report questionnaires, BAASIS was primarily used in the included studies, which was specific for adherence to immunosuppressive therapy and administration. As the number of studies included was quite limited, BAASIS in this meta-analysis did not detect any differences in adherence between the two study groups.

With the advent of smartphones and mobile medical devices, mobile health (mHealth) has become a popular method for medical staff to manage patient therapy. Mobile health can serve as an adjuvant method for delivering health education information, sending reminders to patients to take their medicine, and implementing online education. A systematic review indicated that information technology-based interventions such as mobile health/personal digital assistants (PDAs), computer systems, and multi-components have the potential to improve self-management in adolescents and young adult kidney transplant recipients (Ganjali et al., 2019). However, due to the limited number of studies and absence of sufficient data, our meta-analysis did not find significant results demonstrating that interventions together with mobile health or electronic monitoring were more effective than the routine intervention. The challenging nature of immunosuppressive therapy underscores the need for long-term and persistent interventions.

A combination of multiple interventions may be necessary to maintain adherence. As shown in **Table S1**, adherence-enhancing interventions for immunosuppressive therapy included increasing knowledge related to immunosuppressants, visiting physicians at the appointed times, improving patient behavior, and reminding patients to take their medications in an accurate and timely manner. Our meta-analysis confirmed that interventions led by a multidisciplinary team could improve the immunosuppressive therapy adherence rate for solid organ recipients. Therefore, we believe that a multidisciplinary team approach is a priority for achieving a maximal rate of adherence.

### Limitations

There are limitations to this study that merit consideration. First, although many published studies explored adherence-enhancing interventions on immunosuppressive therapy, only a few published studies exist with RCT designs. This resulted in a limited number of studies with small sample sizes which could be included within our systematic review. Moreover, the outcome data in several of these studies were presented using charts or textual descriptions only, and data that needed to be synthesized or analyzed were unable to be extracted. Second, the exact definition of adherence to immunosuppressants varied among studies, which may have weakened the strength of the evidence garnered. Third, with the exception of the overall adherence rate, subgroup analyses were not included in other outcomes (dosing adherence, timing adherence, and blood tacrolimus level) because of the limited number of studies. Fourth, most of the studies included focused on adherence in kidney recipients, with studies involving other solid organs (liver, lungs, heart, and pancreases) being substantially less represented in these analyses. In this way, it is not possible to provide evidence regarding the effectiveness of adherence-enhancing interventions for liver/lung/heart/pancreases and other combined organ transplantation recipients. Finally, it is also important to note that due to a lack of recent studies using RCTs, some of the included studies in this systematic were published more than 10 years and some almost 20 years ago, resulting in the inclusion of some references which appear quite dated.

## Conclusion

Adherence-enhancing interventions can be considered effective methods of improving adherence to immunosuppressive therapy. We recommend multidisciplinary team-led, life-long, comprehensive interventions together with mobile health for the administration of immunosuppressive therapy to solid organ recipients. For future studies, RCTs with a larger sample size and long-term follow-up are necessary to overcome the shortcomings of current trials. The definitions of adherence and non-adherence should be consistent and clearly described.

## Author Contributions

Y-XS, C-XL, and Y-XF: Made substantial contributions to conception and design, or acquisition of data, or analysis and interpretation of data. Y-XS, C-XL, FL, M-MY, and Y-HJ: Involved in drafting the manuscript or revising it critically for important intellectual content. Y-XS, C-XL, FL, H-MZ, and S-MS: Given final approval of the version to be published. Each author should have participated sufficiently in the work to take public responsibility for appropriate portions of the content. Y-XS, S-MS, and Y-XF: Agreed to be accountable for all aspects of the work in ensuring that questions related to the accuracy or integrity of any part of the work are appropriately investigated and resolved. All authors contributed to the article and approved the submitted version.

## Funding

This work was supported by National Natural Science Foundation of China (No. 71603272 and No. 71974008) and China Postdoctoral Science Foundation (No. 2018M641114 and No. 2020T130029).

## Conflict of Interest

The authors declare that the research was conducted in the absence of any commercial or financial relationships that could be construed as a potential conflict of interest.
