# Supplementary material for: Efficacy of Adherence-Enhancing Interventions for Immunosuppressive Therapy in Solid Organ Transplant Recipients: A Systematic Review and Meta-Analysis Based on Randomized Controlled Trials
Source: Front Pharmacol. 2020 Oct 20;11:578887. doi: 10.3389/fphar.2020.578887 (PMC7606769; doi:10.3389/fphar.2020.578887)
Supplement: Supplementary file 1 [file DataSheet_1.docx]

**Appendix 1. The search strategy in PubMed database**

#1 "Organ Transplantation*"[Title/Abstract]

#2 "Heart Transplantation*"[Title/Abstract]

#3 "Heart-Lung Transplantation*"[Title/Abstract]

#4 "Kidney Transplantation"[Title/Abstract]

#5 "Liver Transplantation"[Title/Abstract]

#6 "Pancreas Transplantation*"[Title/Abstract])

#7 "Renal Transplantation"[Title/Abstract]

#8 "Kidney Grafting"[Title/Abstract]

#9 "Hepatic Transplantation*"[Title/Abstract]

#10 "Pancreas Grafting"[Title/Abstract]

#11 "Liver Grafting"[Title/Abstract]

#12 "Lung Grafting"[Title/Abstract]

#13 "Heart Grafting"[Title/Abstract]

#14 "Cardiac Transplantation*"[Title/Abstract]

#15 "Lung Transplantation"[Mesh]

#16 "Heart-Lung Transplantation"[Mesh]

#17 "Kidney Transplantation"[Mesh])

#18 "Liver Transplantation"[Mesh])

#19 "Pancreas Transplantation"[Mesh]

#20 "Organ Transplantation"[Mesh]

#21 #1 OR #2 OR #3 OR #4 OR #5 OR #6 OR #7 OR #8 OR #9 OR #10 OR #11 OR 12 OR #13 OR #14 OR #15 OR #16 OR #17 OR #18 OR #19 OR #20

#22 Transplantation [Title/Abstract]

#23 liver[Title/Abstract] OR hepatic[Title/Abstract] OR kidney[Title/Abstract] OR renal[Title/Abstract] OR lung[Title/Abstract] OR heart[Title/Abstract] OR Cardiac[Title/Abstract] OR Pancreas[Title/Abstract]

#24 #22 AND #23

**#25** #21 OR #24

#26 Compliance[Title/Abstract]

#27 "Patient Adherence"[Title/Abstract]

#28 "Patient Cooperation"[Title/Abstract]

#29 "Patient Non-Compliance"[Title/Abstract]

#30 "Non Compliance"[Title/Abstract]

#31 Adherence[Title/Abstract]

#32 Nonadherence[Title/Abstract]

#33 Non Adherence[Title/Abstract])

#34 “Medication Adherence” [Mesh]

#35 "Patient Compliance"[Mesh]

**#36** #26 OR #27 OR #28 OR #29 OR #30 OR #31 OR #32 OR #33 OR #34 OR #35

**#37 #25 AND #36**

**TABLE S1 Interventional Strategies of Included Studies**

| **Studies: Authors, Years** | **Intervention period**  **Assessment point** | **Director** | | **Interventional Strategies** | |
| --- | --- | --- | --- | --- | --- |
|  |  | **Intervention** | **Control** | **Intervention group** | **Control** |
| Bessa, 2016 | 8 weeks  Assessed at day 28 and day 90 post-transplantation（Blood TAC trough concentrations obtained at days 10, 14, 21, 28, 60, and 90 after transplant.） | Nurses and 5 pharmacists | Nurses | Standard nursing instructions.  Predefined, specific, standardized, and systematic pharmaceutical care provided by 5 pharmacists.  Visual aids: Pharmacists gave specific, standardized, and systematic instructions that consisted of the importance of the 3 immunosuppressive drugs.  Patients were instructed to always take their immunosuppressive drugs. | Daily standard instructions provided by the nursing staff up to hospital discharge.  The instructions focused on the doses and frequency of immunosuppressive drugs. Patients attended a lecture and received information regarding the care of the surgical wound, importance of all outpatient clinic visits, and the correct use of immunosuppressive drugs.  The nursing staff reinforced the correct use of all medications, but without a specific, standardized, or systematic methodology. |
| Breu-Dejean, 2016 | 8 weeks  Assessed at baseline, 8 weeks and 3 month after the week 8 evaluation) | 1 physician  1 psychologist  2 nurses  1 kinesiotherapist  1 dietician  1 social worker | Not mentioned | Psychoeducational intervention: a multidisciplinary team.  The intervention provided patients with information about their disease. | Standard care. |
| Cukor, 2017 | 2 weeks  12 weeks | Doctoral level psychologists | Not mentioned | Adherence promotion intervention incorporated techniques derived from cognitive behavioral therapy (focused psychotherapy for a wide range of psychological and behavioral issues) and motivational interviewing (a client-centered, semi-directive method of engaging intrinsic motivation to change behavior) techniques.  The sessions were 2 hours long. Three groups of participants completed the two-session intervention and sessions were held between one and two weeks apart. | Participants completed the same number of unannounced telephone pill counts as the intervention condition.  Standard care involved monthly appointments with the nephrology transplant treatment team to assess kidney functioning and address any issues presented by the provider or patient. |
| Chisholm, 2001, USA | 12 months post-transplantation  12 months | Clinical pharmacist and nephrologists | clinical pharmacist and nephrologists | Routine clinical services.  Clinical pharmacy services, which included medication histories and review of patients’ medications and minimizing adverse medication events.  The clinical pharmacist also provided recommendations to nephrologists with the goal of achieving desired outcomes.  The clinical pharmacist counseled patients and instructed them how to properly take their medications. | Routine clinic services.  No clinical pharmacist interaction. |
| Chisholm-Burns, 2013 | 12 months  Assessed at baseline, 3, 6, 9 and 12 months | A trained the study clinical pharmacist | Not mentioned | At the beginning, the lead investigator trained the study’s clinical pharmacist.  In the discussion/negotiation, a toolbox of standardized solutions to adherence barriers was developed as an aid to the contracting process.  Recipients met with the pharmacist to negotiate and sign an adherence contract at baseline, and 3-, 6-, and 9-months post-enrollment. | Standard specialty pharmacy care, which included mail or telephone reminders and an adherence packet consisting of adherence-focused educational pamphlets and a pillbox. |
| Dabbs, 2009 | 2 months  Assessed at 6 months post-transplantation | Transplant coordinator | Transplant coordinator | Standard care.  Pocket PATH: A Pocket PATH device and a user manual, and patients were trained to use the device according to a scripted protocol.  Patients were told to enter data using the device, review data trends using screens and graphs, and follow feedback instructions regarding reporting changes to their transplant coordinator. | Standard care: one-on-one educational session delivered by the transplant coordinator, plus a spirometer and written instructional materials. |
| Dabbs, 2016 | Not available  Assessed at the 2, 6, and 12-month post discharge | The transplant team | Not mentioned | The same discharge instructions that the usual care received.  Patients received a smartphone with custom Pocket PATH programs to record daily health indicators, view graphical displays of trends, and receive automatic feedback messages advising them to notify the transplant coordinator if their health indicators were critical (outside the pre-established parameters).  Training sessions averaged 60 minutes.  A toll-free, technical help line was available. | Received scripted discharge instructions.  An instruction binder that emphasized the importance of performing daily self-management behaviors at home including adhering to elements of the regimen, performing daily self-monitoring, and reporting critical abnormal health indicators to the transplant coordinator based on pre-established parameters. Training sessions averaged 30 minutes. |
| De Geest, 2006 | 3 months  Assessed at 3 months intervention, 6 months follow-up | Nurse | Nurse | Enhanced usual care plus one home visit followed by three follow-up calls, one at the end of the month for three consecutive months. Additional educational, behavioral, and/or social support interventions were conducted during the home visit. | Enhanced usual care: except for home visit and follow-up calls, the other interventions were the same as the intervention group. |
| Dobbels, 2017 | 6 months  5 visits (at inclusion, 3, 6, and 9 and 15months) spread over 15 months | Two nurses with a Master in Nursing Science delivered the intervention and were trained by the study principal investigators | Not mentioned | Theory-based multicomponent-staged tailored medication adherence intervention including social-cognitive and trans-theoretical models.  Motivational interviewing was used as the backbone to deliver the intervention.  An intervention manual was developed that contained an intervention algorithm. Applied interventions were documented on a checklist. | Usual care: used the Helping Hand throughout the study and completed all study visits to control for attention bias (the researcher talked about medication-related topics for 20-30 minutes). |
| Foster, 2018 | 3 months  15 months (3-month run in, followed by a 12-month intervention) | Adherence Support Team included patient, coach and patients’ parent | Coach | Intervention sessions based on the self-management model.  Standardized education on immunosuppressive medications via slide presentations identified adherence barriers via electronic monitoring data and then used action-focused problem-solving to address barriers.  Text messages, emails, or visual cue dose reminders. | Active listening and providing non-specific support. |
| Garcia, 2015 | 3 month  Treatment adherence was assessed at the end of the 3 months | Medical team | Medical team healthcare professional with expertise | Standard education/counseling sessions.  The intervention program was rolled out over 3 months (10 sessions of 30 minutes each) for every study participant delivered on a weekly basis.  The education/counseling sessions covered diverse topics (importance of taking immunosuppressive drugs, a non-judgmental approach to discussing adherence, and tools to integrate medication intake with the patient’s daily routine). | Usual transplant patient education: the immunosuppressant drugs in their first outpatient assessment after discharge.  At each subsequent post-transplant consultation, patients received usual care and advice as part of routine post-transplant care. |
| Geramita, 2020 | Not available  12months (2, 6, and 12 months) | Not mentioned | Not mentioned | Discharge instructions regarding self-management.  A smartphone with the Pocket PATH app with features allowing patients to set reminders for medication-taking and appointments and record and view graphs. | Discharge instructions regarding the self-management transplant program’s standard paper-and-pencil tracking logbook in which patients could record values for the health indicators. |
| Grady, 2019 | 12 weeks  6 months (At baseline and at 3 and 6 months after the initial adult clinic visit) | Pediatric and adult heart transplantation coordinators | Pediatric and adult heart transplantation coordinators | Transition program: approximately 4 months in duration with 2 phases.  During phase 1, at the pediatric site, patients were instructed to complete 4 education modules that focused on knowledge, self-care, and self-advocacy.  Phase 2 began at transfer to adult care and included assessment, reinforcement, and the tailoring of the education module’s content. | A standard transfer of care: patients met with the pediatric coordinator to discuss processes, concerns, and questions regarding transferring care and were asked to schedule a first adult coordinator clinic appointment in 4 weeks. |
| Han, 2019 | 6 months  Assessed at baseline, 28 days, 90 days and 180 days | Coordinators | Coordinators | A medication management smartphone app developed for transplantation patients, audible and/or visual reminders, personal tracking data, patient medication adherence report, information on all immunosuppressants.  An educational video on the importance of immunosuppressant therapy. | At enrollment, patients in both study groups were again educated on the importance of adherence and were taught to take their medication. |
| Harrison, 2017 | 3 months  3 months | Pharmacist | Pharmacist | Standard of care with post-discharge computer-based education.  Subjects in the intervention group received a “learning prescription” tailored to their medication regimen.  Patients had free and unlimited access to complete courses.  Email reminders of educational content aligned with the self-medication program focusing on patient understanding of medications. | The standard of care: pharmacist-led education session, following which patients self-administered immunosuppressants under direct nursing supervision (self-medication program). Teaching occurred in a class of 2 to 5 patients, but may be one on one in selected cases. |
| Hardstaff, 2002 | 3 months  3 months | Nurse Practitioner | Nurse Practitioner | Using smart top bottles: the participants were asked to bring them to their regular outpatient appointments. | Using a pill bottle with an ordinary lid.  Participants brought their pill bottles to their regular outpatient clinic appointments. They were then interviewed about their compliance, and their remaining tablets were counted. |
| Hardstaff, 2003 | Time until feedback ranged from 2 to 6 months  The overall period was 12 months | Nurse practitioner | Not mentioned | Feedback group: received feedback at the first outpatient clinic appointment. Feedback was given after a variable amount of time due to problems collecting data: patients forgot to bring the bottle, left the clinic before being seen by the nurse practitioner to download the information, or their appointment was when she was not available and no one else had access to her computer. | No feedback group: no feedback throughout the course of the trial. |
| Henriksson, 2016 | 12 months  Compliance with immunosuppressive treatment assessed at 1, 2, 3, 4, 5, 6, 7-9 and 10-12 months | Not mentioned | Not mentioned | Electronic medication dispenser: electronic medication dispenser gave visual and audible signals. If the patient did not take their medication, the audible signal was repeated with increasing frequency for 120 minutes. After this (or after the medication was taken), the electronic medication dispenser sent an SMS message to web-based software providing information about patient compliance. | Standard care. |
| Klein, 2009 | 12 months  Assessed at 6 months or12 months | Pharmacist | Not mentioned | Routine clinical care.  Pharmaceutical care services intervention started about 1 week before discharge. The pharmacist met with the patients 3 to 4 times and educated them on immunosuppressive medication. On discharge, the pharmacist handed out and explained written information, including a discharge medication plan. During the first year after transplantation, the patients met the pharmacist at least once per quarter year and at maximum once per month.  The pharmacist discussed with changes in medication, laboratory values, and drug-related problems. Family members were involved. | Routine clinical care |
| Levine, 2019 | 3 months  Assess at 1 and 3 months post transplantation | Not mentioned | Not mentioned | Mobile app user (MAU): a mobile application (app) Transplant Hero was utilized to function as an interactive alarm and remind patients to take their medications as well as provide educational content.  Mobile and watch app users: a wearable smart watch connected through Bluetooth. Pebble Smart watch Technology was used to display the reminder notifications. | No app users. |
| McGillicuddy, 2013 | 3 months  Assessed at pre-intervention and at months 1, 2, and 3 post intervention | Not mentioned | Not mentioned | Using the prototype mHealth system: reminder functions of the medication tray providing instruction on the use of the FORA device and the smartphone.  The reminder functions of the medication tray were enabled. | Standard care: visiting the clinic every 4 to 6 weeks.  Education on all matters related to medical care.  24-hour phone availability of transplant coordinators.  Used medication tray with its reminder functions disabled. |
| Reese, 2017 | 6 months  Assessed at the last 90 days of the trails | Study coordinator nephrologist Pharmacist | Pharmacist | Two intervention groups.  Reminders group: adherence monitoring with customized reminders plus provider notification reminders plus notification group: the percentage of pill bottle-measured adherence was calculated every 2 weeks by Way to Health. If adherence decreased to 90%, the study coordinator would telephone the participant. | A wireless pill bottle that provided no alerts and only tracked adherence. |
| Rosenberger, 2017 | Not available  1 year after transplantation (Interviewed at 2, 6 and 12 months after discharge) | Transplant coordinator | Transplant coordinator | Pocket PATH app: received a smartphone loaded with custom Pocket PATH features. Recorded daily health indicators, provided graphical displays of trends, advised patients to notify coordinator if health indicators outside of pre-established parameters. | Usual care: discharge instructions regarding self-management. |
| Russell, 2011 | 6 months  Assessed at 6 months | Investigator | Nurse | Continuous self-improvement intervention focused on changing the systems in which the person lives using the plan-do-check-act process during the initial home visit and reviewed each month during the 6-month treatment group intervention. | Control intervention: each month during the 6-month intervention phase, patients were provided educational brochures. The first brochure was delivered via a home visit with subsequent brochures mailed. Monthly telephone calls were made. |
| Russell, 2020, USA | 6 months  Assessed at 6 months intervention phase | A trained Baccalaureate‐prepared registered nurse | Registered nurse | The System CHANGE intervention supports patient‐designed, registered nurse interventionist‐guided, small experiments using Deming's plan‐do‐check‐act cycle to redesign the personal environmental system and daily health behavior routines. | Attention control interventions: an in‐person visit where the first of six educational brochures was reviewed.  Subsequently each month for 6 months, the registered nurse contacted the participants to discuss one of the materials. |
| Schmid, 2017 | Not available  Assessed at 0, 3, 6, 12 months post transplantation, | A transplant nurse case manager and two senior transplant physicians  (surgeon and nephrologist) | transplant  nurse  physicians | Standard of care.  Telemedically supported case-management: a tailored telemedically supported case-management model that included three basic components:  (i) A chronic case-management process for the first year post-transplant, (ii) a case-management process applicable for acute care situations, and (iii) a telemedically equipped team.  An interdisciplinary case-management team of transplant center experts delivered the intervention.  Materials: 1 internal server provision including 1 security token, 1 laptop, and 1 mobile phone; touchscreen terminals including software licenses. | Standard of care: prior to discharge, all participants received a booklet for recording drug regimens, vital signs, and fluid balance.  Nurse provided counseling, including self-management information about disease prevention, immunosuppression adherence, and self-monitoring.  Regular checkups.  The physicians determined the time intervals between checkups and offered further consultations whenever needed.  Additional checkups with other specialists were advocated when appropriate. |
| Suhling, 2014 | 6 months  6months (Follow-up was 6 months after start of the education) | Lung-transplant specialists | a trained nurse | Tablet PC education: an iPad was used for education.  A Keynote presentation (Apple) consisting of 30 slides and 4 video clips totaling 12:45 minutes were included. Educational material was mainly paper- and computer-based presentations. | Nurse-led education: the designated written material provided patient instructions. Educational material was mainly paper- and computer-based presentations. Educational content comprised of highlighting the importance of regular medication and side effects. |

**TABLE S2 The outcomes about adherence rate and adherence score in the studies**

| **Studies: Authors, Years** | **Outcomes** | **Assessment method** | | | **Reported assessment points** | **Results from the original studies** |
| --- | --- | --- | --- | --- | --- | --- |
|  |  | **Eletronic monitor** | **Pill account** | **Self report or collateral report** |  |  |
| **Overall adherence rate** | |  |  |  |  |  |
| Cukor, 2017 | Adherence percentage |  | √ |  | Mean adherence of 6 weeks | The intervention group displayed significantly higher levels of adherence when compared to the control group |
| Han, 2019 | The overall adherence rate | √ |  |  | Assessed at baseline, 28 days, 90 days and 180 days | There was no between-group difference |
| Klein, 2009 | Missed one or more doses during the last 4 weeks |  |  | √ | Assessed at 6 months or12 months after discharge | There was no between-group difference |
| Hardstaff, 2002 | 100% adherence | √ |  |  | The remaining tablets were counted at 3-month intervals | unavailable |
| Harrison, 2017 | Total number of doses missed >10% |  |  | √ | Last 7 days of during 3 follow up months | There was no between-group difference |
| Reese, 2017 | Mean adherence rate | √ |  |  | Mean adherence rate at the last 90 days of the trial | Adherence in the reminders group and the reminders-plus-notification group was significantly higher than in the control group |
| Rosenberger, 2017 | Comprehensive adherence rate (including immunosuppressant, attending clinic appointment, monitoring vital signs and monitoring spirometry) |  |  | √ | Unclear | Recipients in intervention group were more likely than control group to be persistent high adherers |
| Schmid, 2017 | Comprehensive adherence rate (combination of tacrolimus trough level, collateral report and self-report) |  |  | √ | At 0, 3, 6, 12 months post transplantation, but the time reported result was unclear | Intervention group significantly higher than control group. |
| Suhling, 2014 | Physicians’ judgment of adherence |  |  | √ | 6-months post intervention | There was no between-group difference |
| **Taking adherence** | |  |  |  |  |  |
| De Geest, 2006 | Taking adherence rate | √ |  |  | Assessed at 3, 6 and 9 months post intervention | There was no between-group difference |
| Foster, 2018 | 100% Taking adherence | √ |  |  | Mean days of taking adherence during 12-month intervention interval | Intervention group significantly higher than control group. |
| Han, 2019 | Taking adherence | √ |  |  | 6-month cumulative adherence rate | There was no between-group difference |
| Klein, 2009 | Mean Taking compliance | √ |  |  | Mean adherence of 12-month | There was no between-group difference |
| Hardstaff, 2003 | Taking adherence | √ |  |  | Overall in the 12 months | unavailable |
| **Dosing adherence** | |  |  |  |  |  |
| Chisholm, 2001 | Dosing compliance rate |  |  |  | Mean of 12 months post-transplantation | Intervention group significantly higher than control group. |
| Chisholm-Burns, 2013 | Dosing compliance rate |  |  |  | Assessed at baseline, 3, 6, 9 and 12 months | The intervention group had significantly higher timing adhenrence rate than control group at 6, 9 and 12 month follow-up period |
| Dobbels, 2017 | Dosing compliance rate | √ |  |  | Assessed at 3-month run-in period, 6-month intervention period and 6-month follow-up period | The intervention group had significantly higher timing adherence rate than control group at 6-month intervention period and 6-month follow-up period |
| Han, 2019 | Dosing adherence | √ |  |  | 6-month cumulative adherence rate | There was no between-group difference |
| Hardstaff, 2003 | Dosing adherence | √ |  |  | Overall in the 12 months | Unavailable |
| Klein, 2009 | Dosing adherence | √ |  |  | Mean adherence of 12-month | Intervention group significantly higher than control group. |
| Reese, 2017 | Dosing compliance rate | √ |  |  | Assessed at the final 90 days of the intervention period | this percentage did not differ across arms |
| **Timing adherence** | |  |  |  |  |  |
| De Geest, 2006 | Timing adherence rate | √ |  |  | Assessed at 3, 6 and 9 months post intervention | There was no between-group difference |
| Dobbels, 2017 | Timing adherence rate | √ |  |  | Assessed at 3-month run-in period(before randomization), 6-month intervention period and 6-month follow-up period | The intervention group had significantly higher timing adhenrence rate than control group at 6-month intervention period and 6-month follow-up period |
| Foster, 2019 | Timing adherence rate | √ |  |  | Mean days of taking adherence during 12-month intervention interval | The intervention group had significantly higher timing adhenrence rate than control group |
| Foster, 2019 | Timing adherence rate |  |  | √ | Mean self-reported adherence during 12-month intervention interval | Self-reported timing adherence did not differ between groups |
| Han, 2019 | Timing adherence rate | √ |  |  | 6-month cumulative adherence rate | There was no between-group difference |
| Klein, 2009 | Timing adherence rate | √ |  |  | Mean adherence of 12-month | There was no between-group difference |
| Harrison, 2017 | Timing adherence rate |  |  | √ | at 3 months. | There was no between-group difference |
| **Adherence score** | |  |  |  |  |  |
| Garcia, 2015 |  |  |  | √ | Assessed at the end of 3 months | Intervention group significantly higher than control group. |
| McGillicuddy, 2013 | Medication adherence score | √ |  |  | Assessed at baseline, 1, 2 and 3 months | Compared to the standard care control group, the intervention group exhibited significant improvements in medication adherence |
| Russell, 2011 | Medication adherence score | √ |  |  | Assessed at baseline, 1, 2, 3, 4, 5 and 6 months | There was a statistically significant difference between groups over the entire six-month period |
| Russell, 2020 | Medication adherence score | √ |  |  | Assessed at baseline, 6 and 12 months | There was a statistically significant difference between groups over the 6 month intervention phase and 6 month maintenance phase |

**TABLE S3 The adherence assessed by self-report questionnaire in the studies**

| **Studies: Authors, Years** | **Name of questionnaire** | **Reported assessment points** | **Results from the original studies** |
| --- | --- | --- | --- |
| Bessa, 2016 | BAASIS | Assessed at day 28, day 90 | There was no between-group difference on these 2 points |
| Dabbs, 2009 | The Health Habits Assessment: determine post-transplant adherence in 10 areas: attending clinic appointments; completing blood work; monitoring home blood pressure and taking the primary immunosuppressant, etc. | Assessed at the first two months following hospital discharge | Intervention group reported significantly higher levels of perceived self-care agency than control group |
| Dabbs, 2016 | The Health Habits Survey was used to assess adherence to all elements of the medical regimen (e.g., taking medications, attending clinic appointments, completing lab work) | Assessed at 2, 6 and 12 months | The intervention group was more likely to show high adherence than the control group |
| Dobbels, 2017 | BAASIS | Unavailable | There was no between-group difference |
| Foster, 2018 | MAM-MM: Medical Adherence Measure Medication Module | Mean self-reported adherence during 12-month intervention interval | Self-reported adherence did not differ between groups. |
| Garcia, 2015 | ITAS:Immunosuppressant Therapy Adherence Scale | Assessed at the end of 3 months | Intervention group significantly higher than control group. |
| Geramita, 2020 | The Health Habits Survey was used to assess adherence to all elements of the medical regimen (e.g., taking medications, attending clinic appointments, completing lab work) | Primary immunosuppressant medication (missed > once per month,%) at 12 months and long term follow up | There was no between-group difference |
| Han, 2019 | BAASIS | Assessed at day 28, day 90 and 180 | There was no between-group difference on these 3 points |
| Suhling, 2014 | BAASIS | Assessed at 6-months post intervention | No differences between inclusion and at 6 months in either group |
| Schmid, 2017 | BAASIS | Assessed at 0, 3, 6, 12 months post transplantation | Intervention group significantly higher than control group. |
| Reese, 2017 | BAASIS | Assessed at the end of the trial | There were no significant differences across arms |
| Breu-Dejean, 2016 | Adherence rate assessed by a French questionnaire | Assessed at baseline, at 8 weeks later and at 3 months after the week 8 evaluation | The data only from the third evaluation was avalible and intervention group had a significantly higher adherence rate than control group |
| Grady, 2019 | 15-item questionnaire measuring adherence to 15 aspects of the medical regimen, including medications [eg, immunosuppressants], lifestyle [eg, diet and exercise], appointment keeping [eg, clinic attendance], and health monitoring [eg, monitoring symptoms] | Average overall self-reported adherence score to the treatment regimen. Data are reported at 0, 3, 6, and 12 months posttransplant | There were no significant differences across arms |

Note: BAASIS: Basel Assessment of Adherence to Immunosuppressive Medication Scale;

MAM-MM: Medical Adherence Measure Medication Module; ITAS:Immunosuppressant Therapy Adherence Scale

**TABLE S4 The outcomes about blood immunosuppressant level in the studies**

| **Studies: Authors, Years** | **Outcomes** | **Reported assessment points** | **Results from the original studies** |
| --- | --- | --- | --- |
| **Tacrolimus concentration** |  |  |  |
| Bessa, 2016 | Mean Tacrolimus trough levels, ng/mL | Assessed at days7, 10, 14, 21, 28, 60, and 90 after transplant | There were no significant differences across arms |
| Cukor, 2018 | Mean Tacrolimus trough levels,ug/L | Tacrolimus whole-blood samples used for the determination of 12h tacrolimus trough concentrations | Mean tacrolimus trough levels did not differ significantly between groups |
| Grady, 2019 | Mean TAC levels | Assessed at baseline, 3 and 6 months post transfer | Mean tacrolimus levels did not differ significantly between groups |
| Garcia, 2015 | Mean Tacrolimus trough levels, ng/dl | Assessed at each outpatient visit up to the 3 months, 6 months and 1 year. |  |
| Reese, 2017 | Mean Tacrolimus trough levels, ug/L | Mean adherence rate at the last 90 days of the trial | There were no significant differences across arms |
| Schmid, 2017 | Tacrolimus trough level (ng/mL) | At 0, 3, 6, 12 months post transplantation | Specific data was not avalible because of comprehensive adherence rate (combination of tacrolimus trough level, collateral report and self-report) |
| **The coefficient of variation for tacrolimus (% CV)** | |  |  |
| Bessa, 2016 | The coefficient of variation for Tacrolimus (% CV) | Mean of CV was caculated from 6 dose-corrected tacrolimus whole blood trough concentrations obtained at days 10, 14, 21, 28, 60, and 90 after transplant | There was no difference between the groups |
| Reese, 2017 | The CVs for tacrolimus level | Assessed at the last 90 days of the trial | There were no significant differences across arms |
| Levine, 2019 | The CVs for tacrolimus level (%) | Assessed at 1 and 3-month post-transplant | There were no significant differences across arms |
| **Within** **immunosuppressant concentration target** | |  |  |
| Bessa, 2016 | Within Tacrolimus target, n (%) | Assessed at days7, 10, 14, 21, 28, 60, and 90 after transplant | There were no significant differences across arms |
| Chisholm, 2001 | Within TAC/cyclosporine target range, n (%) | the time point was unclear | The intervention group had a significantly higher "target rate" than control group |
| Grady, 2019 | Within Tacrolimus target, n (%) | Assessed at baseline, 3 and 6 months post transfer | The frequency was higher in the intervention group than in the usual care group at 3 months and 6 months. |
| Harrison, 2017 | Within Tacrolimus target, n (%) Patients with levels < 5 or > 17, n (%) | Assessed at 3 months. | There was no between-group difference |
| Harrison, 2017 | Within Cyclosporine (C2 monitoring) target (%) <500 or >1500 | Assessed at 3 months. | There was no between-group difference |
| Harrison, 2017 | Within Cyclosporine (C0 monitoring) target (%) <150 or >400 | Assessed at 3 months. | There was no between-group difference |
| Klein, 2009 | Percent of participants within Tacrolimus target, n (%) | From month 2 to 12 posttransplantation | The intervention group had a significantly higher rate of within tacrolimus target than control group |
| Reese, 2017 | Within Tacrolimus target (Mean) | Assessed at the last 90 days of the trial | There were no significant differences across arms |
| Suhling, 2014 | Levels of immunosuppression in target range, % (IQR) | Assessed at 6 months post intervention | There was no difference between the groups |
| **Above immunosuppressant concentration target** | |  |  |
| Bessa, 2021 | Above Tacrolimus target, n (%) | Assessed at days 7, 10, 14, 21, 28, 60, and 90 after transplant | There were no significant differences across arms |
| Klein, 2009 | Percent of participants above tacrolimus target, n (%) | From month 2 to 12 posttransplantation | The control group had a significantly higher rate of above tacrolimus target than intervention group |
| **Below tacrolimus target** | |  |  |
| Bessa, 2019 | Below tacrolimus target, n (%) | Assessed at days 7, 10, 14, 21, 28, 60, and 90 after transplant | There were no significant differences across arms |
| Klein, 2009 | Percent of participants below tacrolimus target, n (%) | From month 2 to 12 posttransplantation | The control group had a significantly higher rate of above tacrolimus target than intervention group |
| **Standard deviation (SD) of immunosuppressant concentration** | |  |  |
| Foster, 2020 | Standard deviation (SD) of tacrolimus levels (2x/d formulation) | Mean days of taking adherence during 12-month intervention interval | There was no difference between groups in the SD of tacrolimus trough levels |
| Foster, 2021 | Standard deviation of tacrolimus levels (1x/d formulation) | Mean days of taking adherence during 12-month intervention interval | There was no difference between groups in the SD of tacrolimus trough levels |
| Harrison, 2017 | Patients with SD ≥ 3, n (%) | Assessed at 3 months | There was no between-group difference |
| Harrison, 2017 | SD of Cyclosporine (C2 monitoring) ≥ 400, n (%) | Assessed at 3 months | There was no between-group difference |
| Harrison, 2017 | SD of Cyclosporine (C0 monitoring) ≥ 150, n (%) | Assessed at 3 months | There was no between-group difference |


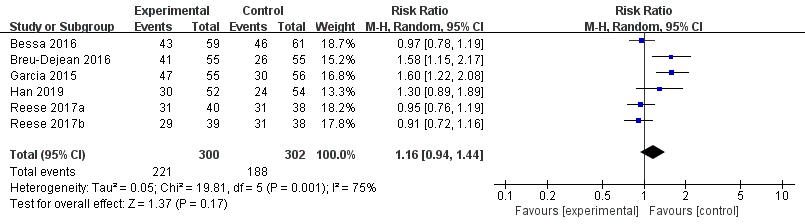


Figure S1. Forest plot of adherence rate by self-report questionnaire. Risk Ratio (RR) with 95% confidence interval (CI) between the adherence enhancing intervention group and routine intervention groups


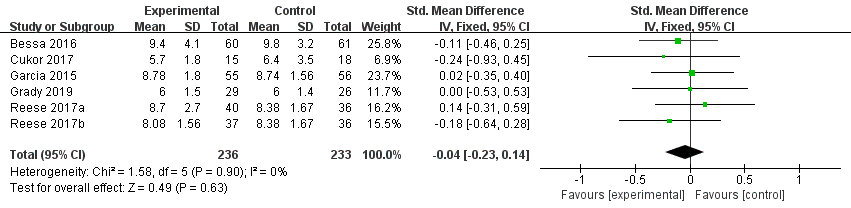


Figure S2. Forest plot of tacrolimus level. Standardized mean difference (SMD) with 95% confidence interval (CI) between the adherence enhancing intervention group and routine intervention groups


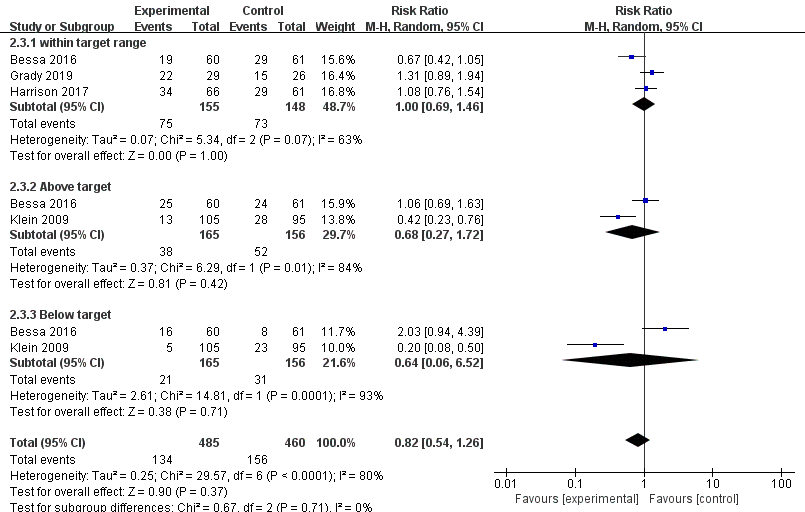


Figure S3. Forest plot of blood immunosuppressant level in different range. Risk Ratio (RR) with 95% confidence interval (CI) between the adherence enhancing intervention group and routine intervention groups


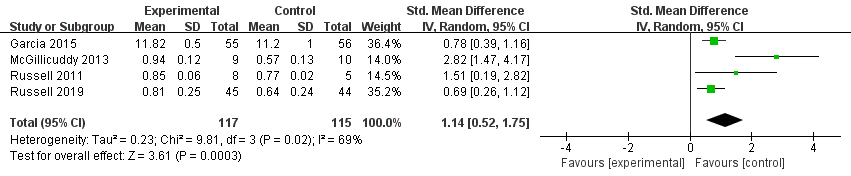


Figure S4. Forest plot of adherence score. Standardized mean difference (SMD) with 95% confidence interval (CI) between the adherence enhancing intervention group and routine intervention groups


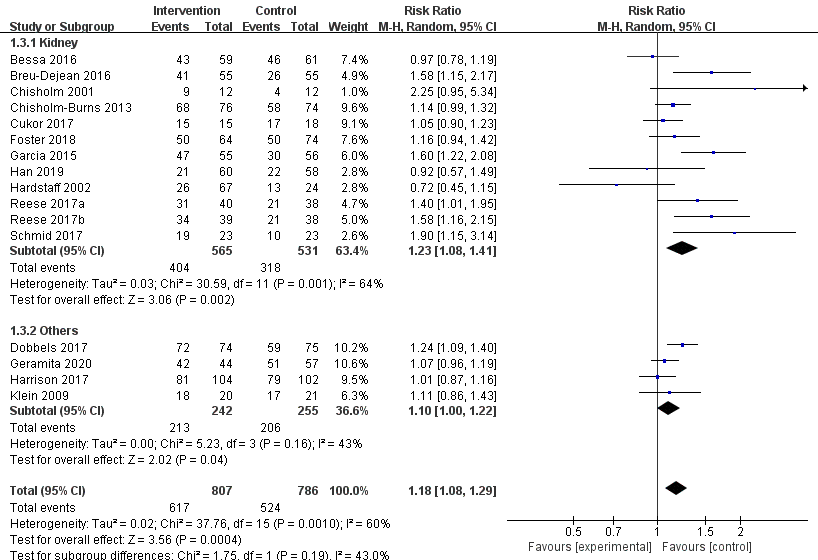


Figure S5. Forest plot of overall adherence rate (subgroup analysis by organ type). Risk Ratio (RR) with 95% confidence interval (CI) between the adherence enhancing intervention group and routine intervention groups


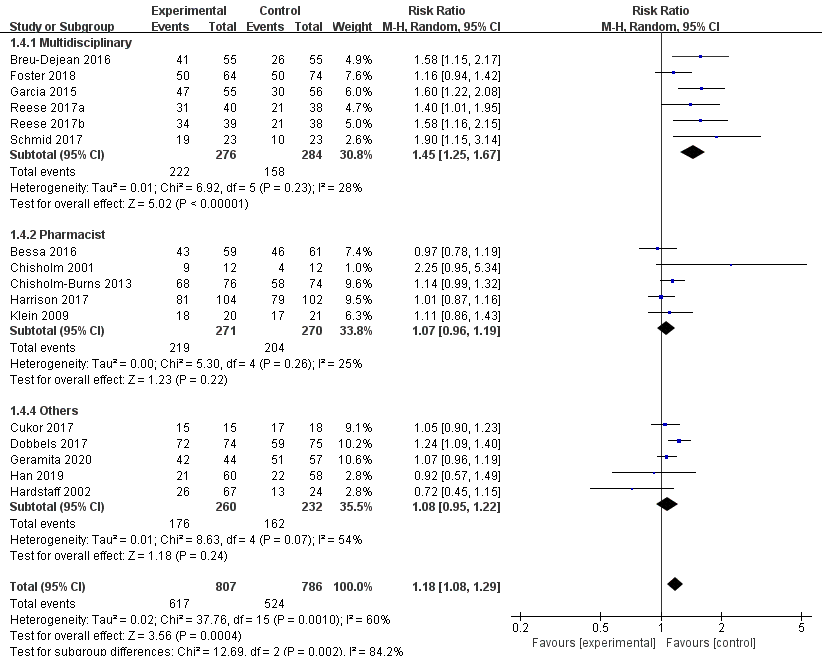


Figure S6. Forest plot of overall adherence rate (subgroup analysis by intervention director). Risk Ratio (RR) with 95% confidence interval (CI) between the adherence enhancing intervention group and routine intervention groups


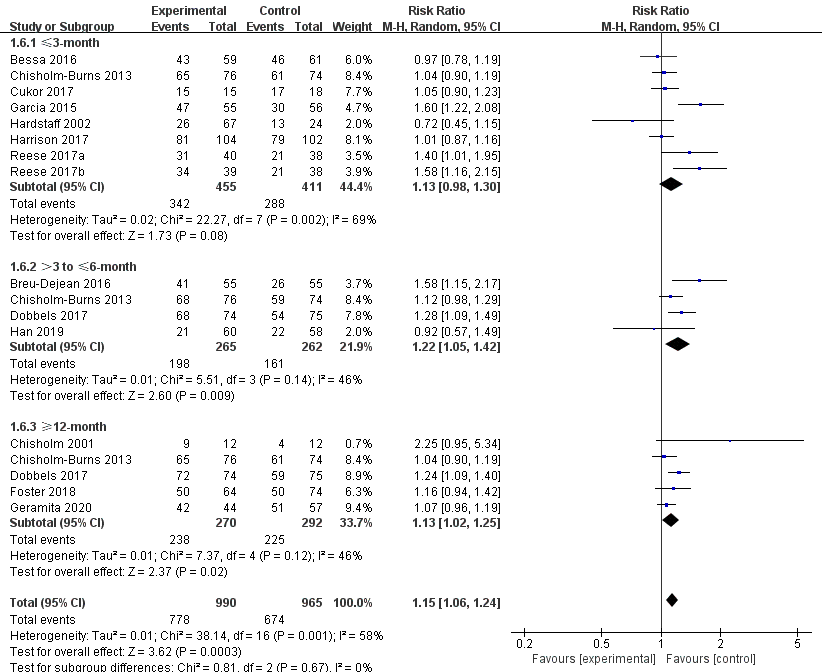


Figure S7. Forest plot of overall adherence rate (subgroup analysis by follow up time). Risk Ratio (RR) with 95% confidence interval (CI) between the adherence enhancing intervention group and routine intervention groups


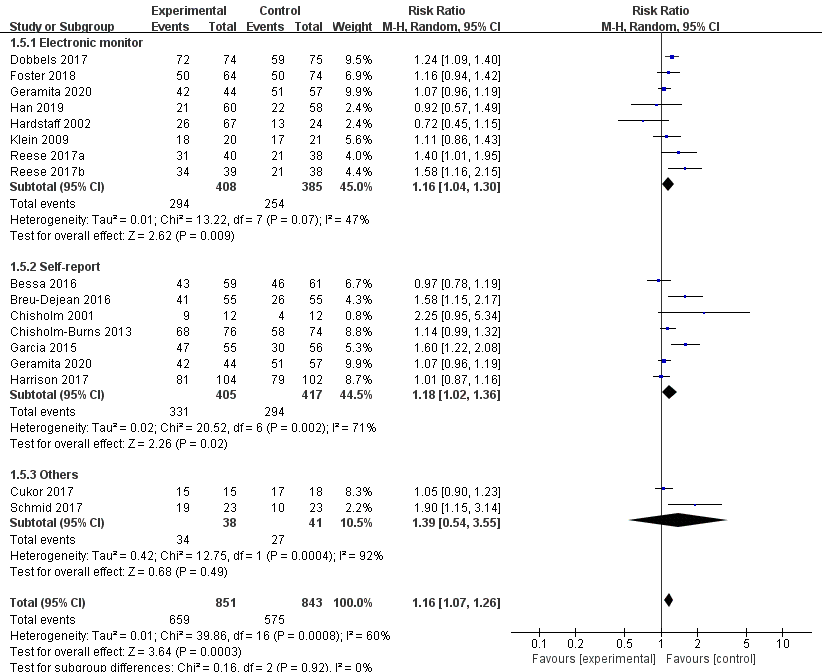


Figure S8. Forest plot of overall adherence rate (subgroup analysis by assessment methods). Risk Ratio (RR) with 95% confidence interval (CI) between the adherence enhancing intervention group and routine intervention groups


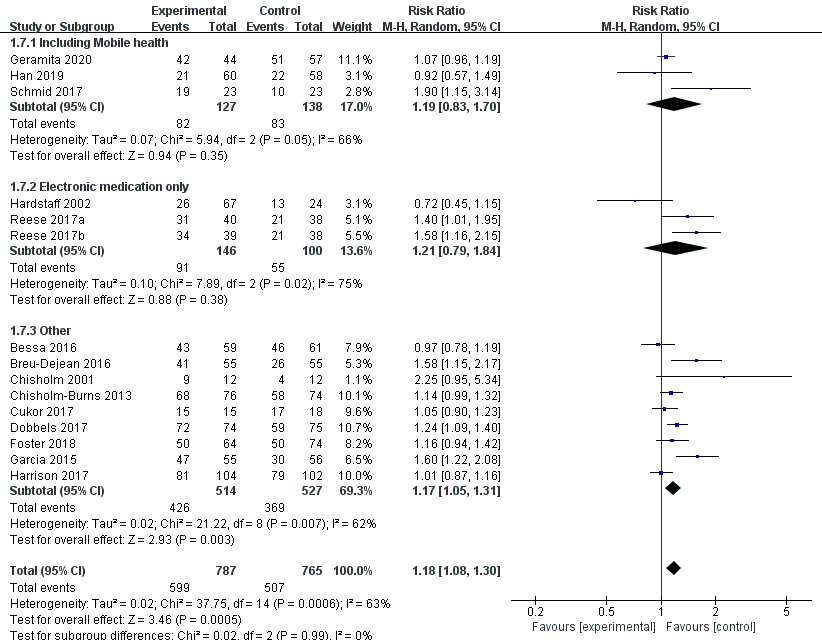


Figure S9. Forest plot of overall adherence rate (subgroup analysis by intervention methods). Risk Ratio (RR) with 95% confidence interval (CI) between the adherence enhancing intervention group and routine intervention groups
